# Knowledge, Attitudes, and Practices of Urban Residents Toward COVID-19 in Shaanxi During the Post-lockdown Period

**DOI:** 10.3389/fpubh.2021.659797

**Published:** 2021-05-20

**Authors:** Na Xu, Yongai Zhang, Xiaona Zhang, Guangwei Zhang, Zikai Guo, Nan Zhao, Fei Li

**Affiliations:** ^1^School of Nursing, Xi'an Medical University, Xi'an, China; ^2^Department of Basic Medicine, Xi'an Medical University, Xi'an, China; ^3^Center for Medical Language and Culture Studies, Xi'an Medical University, Xi'an, China; ^4^Department of Public Health, Xi'an Medical University, Xi'an, China

**Keywords:** knowledge, attitude, practices, residents, COVID-19

## Abstract

**Background:** The coronavirus disease 2019 (COVID-19) pandemic in China is essentially under control. Under global scrutiny, China has started reviving the social, cultural, and working lives of its inhabitants. However, localized outbreaks of COVID-19 are occurring, indicating that the country still needs to follow disease prevention and control measures. Previous studies have assessed the knowledge, attitudes, and behaviors of the general public in China regarding COVID-19 during the pandemic. However, little is known about knowledge, attitudes, and practices (KAP) of Chinese residents regarding COVID-19 after periods of lockdown. Therefore, this study was conducted to identify the KAP and other factors among the residents of Shaanxi Province during the post-lockdown period.

**Methods:** A cross-sectional, network questionnaire survey was conducted in Shaanxi Province from October 1–30, 2020. A total of 1,175 urban residents were interviewed via Wen Juan Xing, an online questionnaire tool. A self-developed online KAP COVID-19 questionnaire was developed in this study. The questionnaire consisted of four parts: general information, knowledge, attitude, and practice. Descriptive statistics and binomial logistic regression analysis were used in the statistical analysis.

**Results:** The majority of the participants were knowledgeable about COVID-19. They had optimistic attitudes and behaved appropriately toward COVID-19. Education was an associated factor for the knowledge of residents and the knowledge of COVID-19 was high among people with high academic qualifications. Attitudes were more positive in residents who lived with elderly people, women, and children. The score of practice was higher in residents with positive attitudes and high academic qualifications. There was a positive correlation between age and practice.

**Conclusion:** We found that the KAP of residents in Shaanxi was at a relatively high level during the post-lockdown period. Although the lockdown was lifted, the epidemic is not over. Thus, it is necessary to develop targeted health education programs for residents with different demographic characteristics in Shaanxi.

## Introduction

The World Health Organization (WHO) officially declared the coronavirus disease 2019 (COVID-19) a public health emergency of international concern on January 30, 2020 (1). As of April 20, 2021, there have been 141,549,845 laboratory-confirmed cases of COVID-19, including 3,021,397 deaths in 223 countries, territories, and areas (2). In order to control the COVID-19 epidemic in China, the Wuhan government imposed a lockdown starting on January 23, 2020, and the residents were required to stay at home to self-isolate (3). Other cities subsequently imposed a series of measures to control the epidemic (4). On April 8, 2020, Wuhan, which once attracted worldwide attention because of the severe epidemic situation, reopened its transportation connections to areas outside the city. This marked a major victory in China's war against the COVID-19 epidemic (3). Currently, China has begun to resume production (5), and people in China have gradually returned to their normal life. However, localized outbreaks of COVID-19 are still occurring in some places in China, such as Beijing, Xinjiang, the Inner Mongolia Autonomous Region, and Shanghai (6–9). This indicates that the country still needs to follow disease prevention and control measures.

The knowledge, attitudes, practices (KAP) model is a common model used to explain how individual knowledge and attitudes affect healthy behavior changes. It was proposed by Mayo, a professor at Harvard University in the 1960s. It is one of the basic patterns of changing human health behavior. The KAP model assumes that the occurrence of healthy behaviors can be divided into three continuous processes. The first is the correct perception of health knowledge by the individual. On this basis, the individual establishes positive beliefs and attitudes, and finally it is possible to form a healthy behavior. The term “knowledge” refers to the understanding of disease-related knowledge. “Attitudes” refers to the trust in the relevant knowledge that has been acquired. “Practices” refers to the generation of behaviors that are conducive to health under the basis of mastering health knowledge and the promotion of health beliefs (10). In summary, knowledge is the basis for changing behaviors, and attitudes are the driving force for changing behaviors. According to the KAP model only by acquiring health-related knowledge and thinking repeatedly, and only by raising knowledge to belief, can it be possible to adopt a positive attitude to produce healthy behavior. Research has shown that people's knowledge, attitudes and practices toward COVID-19 are important determinants of whether they participate in pandemic-specific prevention behaviors. Scholars believe that an elevated KAP toward COVID-19 preventive measures must be related to higher commitment in appropriate defensive behavior during the pandemic (11). Numerous empirical studies have demonstrated that health education can improve knowledge and change unfavorable attitudes and behaviors, effectively curbing infectious diseases and epidemics (12). Therefore, understanding people's knowledge, attitudes, and behaviors toward COVID-19 can provide a reference for the formulation of health education plans. After the outbreak of COVID-19, many researchers have studied the public's knowledge, attitudes, and practices toward COVID-19 in order to provide a basic reference for the formulation of public health education programs. A study conducted in Jordan found that females, aged 40 or older, married, employed, with bachelor's degree or higher, had more knowledge and better practice toward COVID-19 ^1^. Azlan et al. examined the knowledge, attitudes, and practices of the public toward COVID-19 in Malaysia. They found a high knowledge score and a moderate practice score among the participants (13). Roy et al. Assessed attitudes and practices among the adult population in the COVID-19 pandemic of India and found the responders to have a moderate level of knowledge about COVID-19 (14). According to Salman et al., Pakistani university students and employees have good knowledge and attitude regarding COVID-19, but unsatisfactory preventive practices (15). In China, relevant research had also been conducted on the public knowledge, attitude, and behavior about COVID-19 in the early stage of the outbreak. The study conducted by Zhong et al. and Lin et al. showed that the KAP scores of the public in China toward COVID-19 were positive, in particular, the KAP of women who had a relatively high socioeconomic status (16, 17). A study conducted in Anhui, China, found that the residents had a good KAP about COVID-19, yet it was necessary to strengthen the community publicity (18). These studies were conducted at the beginning of the outbreak, and the high level of public knowledge, attitude, and behavior about the epidemic had been confirmed. Although China has begun to return to production and work, there are still sporadic outbreaks, and the campaign against COVID-19 is not over. The fight against COVID-19 is currently ongoing in China. However, little is known about the KAP of Chinese residents regarding COVID-19 during the post-lockdown period. Whether people's knowledge, attitude, and behavior about COVID-19 change in the post-lockdown period requires further research. Thus, it is important to understand the KAP of the Chinese people toward COVID-19 during the post-lockdown period to help determine whether people's knowledge, attitudes, and behaviors about the epidemic have weakened as their lives and economy gradually get on the right track.This will provide a basic reference for the formulation of public health education programs in the new stage.

The aim of this study was to evaluate the KAP regarding COVID-19 among residents in Shaanxi Province, China, and the factors that affect them. Shaanxi Province is located in the northwest of China, connecting the East and the West. It is the starting point of the Silk Road and an important transportation hub for cultural tourism to the Holy Land from China (19). Shaanxi Province, which borders Hubei Province and the Inner Mongolia Autonomous Region, has the largest number of neighboring provinces in China (19). With the recovery of the economy, the population mobility is large, and the task of epidemic prevention and control is arduous. Our study was conducted during the post-lockdown period of the epidemic in Shaanxi Province. Therefore, our study not only provides baseline information on the awareness of the epidemic among residents in Shaanxi but can also assist the authorities in understanding the weaknesses in the epidemic prevention and control program and provide more targeted prevention and control measures during the post-lockdown period.

## Methods

### Study Design and Participants

This was a cross-sectional, descriptive study, which aimed to investigate the KAP regarding COVID-19 risk and the factors that affect them among the local residents in the Shaanxi Province of China during the post-lockdown period. Shaanxi is located in the hinterland of northwest China and consists of 10 cities that are geographically divided into three strata as urban, suburban, and rural regions. From October 1–30 (during the post-lockdown period), five cities (Xi'an, Baoji, Shangluo, Yulin, and Ankang) were selected from Shaanxi Province according to their geographic location. These five cities were distributed in the east, west, north, south, and middle regions of Shaanxi Province. Two communities in each city were randomly selected from the urban regions. Therefore, a total of ten communities were included in this study.

Participants were recruited through purposive and snowball sampling routes from the ten communities. The inclusion criteria were that the age of the subject must be at least 18 years old, and the resident gave consent before completing the survey and the resident could finish the survey by him- or herself. In the pilot testing of 100 people in this study, the standard deviations of knowledge, attitude, and behavior scores were 1.37, 3.99, and 7.31, respectively. The allowable error of the knowledge was not more than 1, α = 0.05, and the required sample size was *n* = ${\left(U_{\text{α}/2}\right)}^{2}$ × 1.37^2^/1^2^ = 8 (20). The allowable error of the attitude and behavior were not more than 2, α = 0.05, and the required sample size was *n* = ${\left(U_{\text{α}/2}\right)}^{2}$ × 3.99^2^/2^2^ = 16, *n* = ${\left(U_{\text{α}/2}\right)}^{2}$ × 7.31^2^/2^2^ = 52 (14). The maximum value of 52 was selected as sample size from one community. Considering the possibility of 20% invalid questionnaires, a minimum of 63 participants were required from each community. In other words, at least 630 participants were required from the 10 communities. We recruited as many participants as possible in our study. This study was approved by the Ethics Committee of Xi'an Medical University (Xi'an, Shaanxi Province, China) (approval NO. XYLS2020135). Before answering the questionnaire, all of the participants had to answer a yes/no question to confirm their willingness to participate in this research voluntarily.

### Study Tools

The questionnaire used was developed by the authors, and the variables regarding the knowledge, attitudes, and practices were based on the previous literature, the recent guidelines of WHO (16–18, 21) and opinions from five experts (one professor of nursing management, one professor of community nursing, one professor of nursing education, one professor of nursing research, and one doctor of epidemiology and statistics). We have already thanked the five experts in the acknowledgment section. The questionnaire consisted of two sections. Section 1 inquired about the demographic data of the residents (six items), which included gender, age, profession, marital status, education, and whether they live with elderly people, women, or children (e.g., children ≤ 5 years old, elderly people ≥ 65 years old, or pregnant women). The professions were divided into manual laborers, white-collar workers, students, and other. The “other” professions included unemployed, retirees, and other professions that were not mentioned. Marital status included married and other, and “other” included single, divorced, and widowed.

Section 2 included questions on the KAP regarding COVID-19 among the residents. The appropriateness of Section 2 of the questionnaire was verified on the basis of the Content Validity Index and was evaluated by the five experts mentioned above. The Content Validity Index of the KAP COVID-19 Questionnaire among the residents was 0.92. Additionally, the validity of the preliminary questionnaire was assessed through pilot testing, which was conducted with 100 residents, and Cronbach's α coefficient of the questionnaire was 0.720. Section 2 was divided into three parts. Part 1 was comprised of 12 items that assessed the knowledge of the residents on COVID-19—source of infection (one item), route of the transmission (one item), epidemicity (one item), symptoms (two items), treatment and vaccines (two items), and prevention measures (five items). The responses were “yes” “no” and “don't know.” Each correct response was scored 1, whereas an incorrect answer and the middle response were scored 0. The total score of the knowledge questionnaire was 12, and higher scores indicated a higher knowledge level of COVID-19. In the pilot study, Cronbach's α coefficient of the knowledge questionnaire was 0.705, and Cronbach's α coefficient of the knowledge questionnaire for the entire study was 0.723. Part 2 consisted of seven items that assessed the attitude of the residents on COVID-19—risk perception of COVID-19 (one item), attitudes toward the government's infection control measures (two items), attitudes toward the people's infection control measures (one item), confidence in other residents' ability to prevent infection (two items), and vaccine acceptability (one item). The answers were evaluated by a 5-point Likert rating scale, ranging from strongly disagree (score 1) to strongly agree (score 5). The score of the attitude questionnaire ranged from 7 to 35, and higher scores indicated a more positive attitude. In the pilot study, Cronbach's α coefficient of the attitude questionnaire was 0.754, and Cronbach's α coefficient of the attitude questionnaire for the entire study was 0.767. Part 3 was comprised of 12 items that assessed the practices of the residents regarding COVID-19—positive coping styles (four items) and defensive coping styles (eight items). Each item was scored 1 (never) to 5 (always). The total score of the practice questionnaire ranged from 12 to 60, and a higher score represented a more positive level of practice. In the pilot study, Cronbach's α coefficient of the practice questionnaire was 0.888, and Cronbach's α coefficient of the practice questionnaire for the entire study was 0.882. We defined when the total score of the KAP questionnaire was higher than P_50_ (the median number of the total score), the level of the KAP of the participants was considered good, and the assigned value was 1; otherwise, the levels were considered to be poor, and the assigned value was 0.

### Data Collection

After the questionnaire was input into the Wen Juan Xing (https://www.wjx.cn/, Wenjuanxing Tech Co. Ltd, Changsha, China), a professional online questionnaire tool, the quick response code of the online questionnaire was formed. The survey was completed during the post-lockdown period. The quick response code of the questionnaire was distributed to the residents who lived in the 10 communities. Then, we asked the voluntary resident participants in the study to send the quick response code to their neighbors in their community. The purpose and instructions of the survey were attached to each questionnaire. The participants had to answer a yes/no question to confirm their willingness to participate voluntarily. After the residents completed all the questions, they could clicked the “submit” button. To prevent multiple answering from the same individual, the questionnaire could only be answered once. All of the data were kept anonymous and confidential throughout the study.

### Statistical Analysis

The collected data were analyzed using SPSS24.0 (IBM, Armonk, NY, USA). Descriptive statistics were used to describe the demographic data, as well as the KAP scores of the residents. The normality of the data was determined by Kolmogorov-Smirnov and Shapiro-Wilk tests. Median and interquartile ranges were measured as the data showed skewed distribution. As noted in the description of the study tools, the KAP was divided into two categories and these were used as dependent variables. The values of the independent variables were as follows: males were assigned 1 and females were assigned 2; married was assigned 1 and others were assigned 2; manual work was assigned 1, white-collar work was assigned 2, others were assigned 3, and student was assigned 4; junior college and below was assigned 1, and bachelor's degree and above was assigned 2; living with the elderly people, women, and children was assigned as 1, and not living with elderly people, women, or children was assigned as 2. Binomial logistic regression analysis used all of the demographic variables as independent variables, and the KAP scores as the outcome variables were conducted to identify factors associated with KAP. Odds ratios (ORs) and their 95% confidence intervals were used to quantify the association between the variables and KAP. *P* < 0.05 was considered as the significant level in this study.

### Results

A total of 1,217 questionnaires were collected through WJX. The questionnaires that were answered with the same option in the knowledge, attitudes, or practices sections were regarded as invalid. Finally, 1,175 effective questionnaires were received. The effective recovery rate was 96.55%. The average age of the participants was (34.50 ± 12.70). The majority of the participants were women (68.1%). Of the participants, white-collar workers were the majority. Married people (59.1%) accounted for the largest group in this study. Among the participants, the group of persons with a bachelor's degree and above was the largest (57.9%), followed by those with a junior college degree or below (42.1%). Thirty-seven percent of the participants lived with susceptible people (Table 1).

**Table 1:** Demographic characteristics of the population surveyed regarding their COVID-19 knowledge, attitudes, and practices (*n* = 1,175).

**Characteristics**	**Mean ± SD**	***N***	**%**
Age (years)	34.50 ± 12.70		
**Gender**			
Male		375	31.9
Female		800	68.1
**Profession**			
Manual workers		350	29.8
White-collar workers		455	38.7
Other^#^		135	11.5
Students		235	20.0
**Marital status**			
Married		695	59.1
Other^*^		480	40.9
**Education**			
Junior college and below		495	42.1
Bachelor's degree and above		680	57.9
**Living with the most susceptible people**			
Yes		435	37.0
No		740	63.0

# *Other professions included unemployed, retirees, and other professions that are not mentioned above*.

* *Other marital statuses included single, divorced, and widowed. COVID-19, coronavirus disease 2019; SD, standard deviation*.

### Knowledge of the Residents Toward COVID-19

The median and average score for the knowledge of the residents regarding COVID-19 was 10 (*P*_25_ = 9, *P*_75_ = 11; 9.51 ± 1.49), with a range of 0–12. The rate of correct answer of the 12 questions on the knowledge section was between 31.9 and 99.1%. Among the items regarding the knowledge of COVID-19, the three items with the highest rate of correct answers were items 2, 9, and 10, and those with the lowest rate were items 11, 7, and 6 (Table 2).

**Table 2:** Knowledge of community residents regarding COVID-19 (*n* = 1,175).

**Knowledge Statement**	**Correct, N**	**(%)**
Asymptomatic infection is not infectious, and there is no risk of transmission. (False)	1,085	92.3
COVID-19 is mainly transmitted through respiratory droplets and close contact transmission. (True)	1,165	99.1
The prevalence of COVID-19 is not seasonal. Whenever there is no adequate control measures, there will always be a prevailing risk. (True)	1,085	92.3
Fever is one of the main symptoms of COVID-19. (True)	1,055	89.8
Coughing is one of the main symptoms of COVID-19. (True)	885	75.3
At present, specific drugs have been developed for COVID-19. (False)	670	57.0
China has approved two vaccines for emergency use in June 2020, and officially launched the emergency use of a COVID-19 vaccine in July. (True)	505	43.0
The public does not need to wear masks when they are at home, outdoors, in areas without people gathering, and in areas with good ventilation. (True)	1,030	87.7
Masks can be worn on both sides, regardless of the front and back. (False)	1,150	97.9
Wash hands with running water after going to the toilet, before eating, after going out, before holding children, and after touching animals. (True)	1,145	97.4
Hand disinfectant without washing can be used as a routine method of hand cleaning. (False)	375	31.9
Fumigating rooms with vinegar can prevent COVID-19. (False)	1,025	87.2
**Total: Mean ± SD:** 9.51 ± 1.49**; M (P**_**25**_**, P**_**75**_**):** 10 (9, 11)	–	–

*COVID-19, coronavirus disease 2019; M, median; SD, standard deviation*.

### Attitude of the Residents Toward COVID-19

The median and average score of the attitudes toward COVID-19 was 30 (*P*_25_ = 27, *P*_75_ = 32; 29.49 ± 3.98) with a range of 7–35. Overall, most of the residents had active attitudes toward COVID-19. In total, 85.4% of the participants agreed that COVID-19 has been effectively controlled in their area. More than half (89.8%) expressed satisfaction with the government's prevention and control measures in this epidemic. Over 85% of the residents believed that the government released information related to COVID-19 in a timely and transparent manner. Over 71% of the subjects thought the people around them had a good protection for COVID-19. More than 95% of the participants believed that they would definitely be able to overcome this epidemic (Table 3).

**Table 3:** Attitudes of community residents regarding COVID-19 (*n* = 1,175).

**Attitude Statement**	**Mean ± SD**	**M (P_**25**_, P_**75**_)**	***N*** **(%)**				
			**Strongly disagree**	**Disagree**	**Neutral**	**Agree**	**Strongly agree**
I think COVID-19 has been effectively controlled in my area.	4.08 ± 0.97	4 (4, 5)	40 (3.4)	65 (5.5)	75 (6.4)	575 (48.9)	420 (35.8)
I am satisfied with the measures taken to prevent and control the COVID-19 epidemic in my place.	4.35 ± 0.79	4 (4, 5)	15 (1.3)	20 (1.7)	85 (7.2)	470 (40.0)	585 (49.8)
I think the government can timely and transparently release information related to COVID-19.	4.29 ± 0.84	4 (4, 5)	15 (1.3)	15 (1.3)	155 (13.2)	425 (36.2)	565 (48.0)
I think the people around us have good protection for COVID-19.	3.87 ± 0.87	4 (3, 4)	20 (1.7)	40 (3.4)	290 (24.7)	545 (46.4)	280 (23.8)
I believe we will definitely be able to overcome the COVID-19 epidemic.	4.75 ± 0.64	5 (5, 5)	15 (1.3)	0 (0)	40 (3.4)	150 (12.8)	970 (82.5)
I believe that as long as I do a good job in self-protection, I will not be infected with COVID-19.	3.94 ± 1.08	4 (4, 5)	55 (4.7)	85 (7.2)	140 (11.9)	495 (42.1)	400 (34.1)
If there is a vaccine against COVID-19, I will take it.	4.20 ± 0.89	4 (4, 5)	25 (2.1)	10 (0.9)	190 (16.2)	425 (36.2)	525 (44.6)
Total	29.49 ± 3.98	30 (27, 32)					

*COVID-19, coronavirus disease 2019; SD, standard deviation*.

### Practices of the Residents Against COVID-19

Most of the participants practiced the control measures, as shown in **Table 5**, in a suitable manner. The median and average score of the practices toward COVID-19 was 48 (*P*_25_ = 44, *P*_75_ = 53; 47.77 ± 7.49), with a range of 12–60. There was high compliance (87.2% for “often” and “always”) with wearing face masks to protect themselves when in close contact with others (<1 meter) in crowded places. Over 92% paid attention to hand hygiene and washed hands frequently to prevent infection. Among the questionnaire of practices, items 11, 5, and 10 had the lowest compliance (52.4, 54.0, and 62.2%, respectively) (Table 4).

**Table 4:** Practices of community residents against COVID-19 during the epidemic (*n* = 1,175).

**Practice statement**	**Mean ± SD**	**M (P_**25**_, P_**75**_)**	***N*** **(%)**				
			**Never**	**Seldom**	**Sometimes**	**Often**	**Always**
I take the initiative to learn knowledge related to COVID-19.	3.96 ± 0.89	4 (4, 5)	15 (1.3)	70 (6.0)	190 (16.2)	570 (48.5)	330 (28.1)
I often monitor my body temperature, pay close attention to symptoms, such as fever and cough, and seek medical treatment in time.	3.94 ± 0.98	4 (3, 5)	10 (0.9)	120 (10.2)	175 (14.9)	495 (42.1)	375 (31.9)
I exchange knowledge regarding COVID-19 with my family, relatives, friends, and so on.	3.82 ± 0.93	4 (3, 4)	10 (0.9)	115 (9.8)	220 (18.7)	560 (47.7)	270 (22.9)
I pay close attention to the information regarding the epidemic released by the health department and other official authoritative channels and treat the epidemic situation rationally.	4.15 ± 0.84	4 (4, 5)	10 (0.9)	40 (3.4)	160 (13.6)	520 (44.3)	445 (37.8)
I perform the appropriate physical exercises at home to prevent the disease.	3.53 ± 1.06	4 (3, 4)	20 (1.7)	215 (18.3)	305 (26.0)	395 (33.6)	240 (20.4)
I will carry a mask with me, and I will wear a mask when I am in close contact with others (<1 meter) in crowded places.	4.34 ± 0.77	4 (4, 5)	10 (0.9)	5 (0.4)	135 (11.5)	450 (38.3)	575 (48.9)
I always pay attention to hand hygiene and wash my hands frequently to prevent COVID-19.	4.48 ± 0.72	4 (5, 5)	5 (0.4)	20 (1.7)	65 (5.5)	405 (34.5)	680 (57.9)
I pay attention to ventilating rooms to prevent COVID-19.	4.40 ± 0.72	4 (5, 5)	5 (0.4)	20 (1.7)	70 (6.0)	490 (41.7)	590 (50.2)
I have reduced the frequency of dining out and dining together.	3.95 ± 0.99	3 (4, 5)	15 (1.3)	95 (8.1)	225 (19.1)	435 (37.0)	405 (34.5)
When I go out or even at home, I have separate meals or use serving chopsticks and spoons when I have group meals with relatives, friends, and colleagues.	3.61 ± 1.18	3 (4, 5)	80 (6.8)	145 (12.3)	220 (18.7)	435 (37.0)	295 (25.2)
No matter where I go (or even at home) and I have meals with my family, I have separate meals or use serving chopsticks and spoons.	3.34 ± 1.29	4 (2, 4)	120 (10.2)	225 (19.1)	215 (18.3)	360 (30.6)	255 (21.8)
I adjust my mood in a variety of ways.	4.24 ± 0.81	4 (4, 5)	5 (0.4)	35 (3.0)	140 (11.9)	485 (41.3)	510 (43.4)
Total	47.77 ± 7.49	48 (44,53)	–	–	–	–	–

*COVID-19, coronavirus disease 2019; SD, standard deviation*.

### Relevant Factors About Knowledge, Attitude and Practice Toward COVID-19

The binomial logistic regression analysis found that the education level of bachelor's degree and above (vs. junior college and below, OR: 3.98, *P* < 0.001) was significantly associated with the participants' knowledge toward COVID-19 (Table 5).

**Table 5:** Binary logistic regression analysis on factors significantly associated with the knowledge, attitudes, and practices toward COVID-19 (*n* = 1,175).

	**Variables**		**OR (95% CI)**	***P***
Knowledge	Education	Junior college and below (reference group)		
		Bachelor's degree and above	3.98 (1.86–8.51)	0.000
Attitudes	Live with elderly people, women, and children	Yes (reference group)		
		No	0.53 (0.30–0.93)	0.027
Practices	Attitudes		1.23 (1.12–1.34)	0.000
	Age		1.06 (1.02–1.11)	0.009
	Education	Junior college and below (reference group)		
		Bachelor's degree and above	2.36 (1.02–5.48)	0.046

*CIs, confidence intervals; COVID-19, coronavirus disease 2019; OR, odds ratio*.

The binomial logistic regression analysis found that not living with the most susceptible people (vs. living with the most susceptible people, OR: 0.53, *P* = 0.027) was significantly associated with the participants' attitude toward COVID-19 (Table 5).

The binomial logistic regression analysis showed that age (OR: 1.06, *P* = 0.009), COVID-19 attitude score (OR: 1.23, *P* < 0.001), and education level of bachelor's degree and above (vs. junior college and below, OR: 2.36, *P* = 0.046) were significantly associated with the participants' practices toward COVID-19 (Table 5).

## Discussion

There have been studies on the KAP of residents in China during the lockdown period (16–18). However, studies on the KAP of residents during the post-lockdown period in China are still lacking. This study has found that the residents of Shaanxi Province, which was adjacent to Hubei, had a high level of KAP toward COVID-19 during the post-lockdown period. We also identified several significant factors associated with the KAP of residents in Shaanxi Province. This evaluation was essential to improve protective practices for preventing a new outbreak of COVID-19 infection among the public in Shaanxi Province.

The accuracy rate of 12 items ranged between 75.3 and 99.1%, with three items, items 6, 7, and 11, falling below this range. WHO suggests that washing hands with soap and water can kill viruses that may be on our hands (22). For the public, it is not recommended to use hand disinfectant as a routine method of hand cleaning. Hand disinfectants should only be used outdoors when there is no way to wash hands with water and soap (21). In our research, 92.4% of the subjects paid attention to hand hygiene and washed their hands frequently to prevent COVID-19, but only 31.9% of subjects thought that hand disinfectant without washing should not be used as a routine method of hand cleaning. These show that although the awareness rate of the importance of hand washing is high, how to wash hands properly still needs further community popularization and education. Less than 50.0% of the participants understand the emergency use and development status of COVID-19 vaccines. This shows that people do not pay enough attention to the vaccine, and the government and media should increase the popularization of vaccine-related COVID-19 knowledge. Only 57.0% of the residents believed that a specific drug for COVID-19 has not been developed yet. This may be related to the lower mortality and higher cure rate as compared with severe acute respiratory syndrome (SARS) (23). In general, the average score of the knowledge for COVID-19 was 9.51 ± 1.49, with a full score of 12. This indicated that the residents in Shaanxi Province had a high awareness of COVID-19, which was the same as the Chinese residents in previous studies (16, 18). During the post-lockdown period of the epidemic, the residents had a high awareness of the epidemic, which was mainly related to the government and the media's strong publicity, as well as people's high attention to the epidemic. Further analysis found that residents with a bachelor's degree and above were more knowledgeable than their counterparts. It was the same as the previous studies (16). Loai et al. and Zhong et al. had both found that the participants' with bachelor's degree and above had a higher mean score on knowledge of COVID-19 than others ^1^. Highly educated people have a higher cognitive level, which may be due to their strong learning ability and ability to obtain information (16). Roy et al. also thought educated people were much more sensitive to the information released by the government and the media (14). In an early survey of KAP about COVID-19 in China, the knowledge scores were significantly correlated with gender, age and marital status (24). But in our research, gender, age and marital status did not significantly influence knowledge. This might be because in Yue's study, majority of the unmarried residents and women had a bachelor's degree or above (24). In our research, people of different marriages and genders have similar educational backgrounds. At the same time, the government has been vigorously disseminating knowledge toward COVID-19 from the early stage to the later stage of the epidemic to improve people's knowledge of COVID-19. All of these may cause changes in related factors that affect knowledge.

Among the items related to attitude, more than 84% of the participants thought that COVID-19 in their area was effectively controlled. This might be due to the adequate response measures taken by the government and the low number of people infected in Shaanxi Province. Since the outbreak of the epidemic, the government of Shaanxi actively facilitated many preventive measures, even during the post-lockdown period (25). Various prevention and control measures of “warding off transmission from overseas, domestic proliferation, and joint prevention and control of people and goods” are fully implemented in Shaanxi. The first is to strengthen the management and control of personnel who come to Shaanxi and return to Shaanxi. Everyone arriving in Shaanxi from overseas or from high-risk areas in China are required to do a 14-day quarantine at a government-designated location, followed by 7-day home quarantine. The second is to increase nucleic acid testing. Efforts should be put on testing the staff and the external environment of foreign-funded enterprises, farmer's markets, and key tourist attractions. The third is to make solid preparations for prevention, control and treatment. Medical institutions at all levels in Shaanxi have been organized to carry out multiple rounds of training such as nucleic acid testing, hospital infection prevention and control, patient medical treatment, and treatment of abnormal vaccination reactions. The fourth is to promote vaccination in an orderly manner. Personnel at border inspection, customs, airport, and medical staff were offered free vaccination, and no serious adverse reactions occurred. The fifth is to carry out comprehensive, all-round and multi-channel publicity. The government has released epidemic prevention and control measures in winter and spring and has promoted propaganda about them, as well as carried out extensive publicity on prevention and control by openning columns in mainstream media (26). At present, it is necessary to show your health quick response (QR) code when entering and exiting any public place in Shaanxi. People should search the health QR code in Alipay or WeChat and then fill out a form with their information. After examination and approval, they will get a health QR code. People are also required to wear masks if using public transportation. As of March 26, 2020, there were 245 cases in Shaanxi, and 242 of these cases completely recovered (27). This made people more confident about the cure rate of this epidemic. Our findings showed that most residents were satisfied with the government's epidemic prevention work, which confirmed findings in Anhui Province (18). The results indicated that the Chinese government's response to major public health events has become progressively mature since the severe acute respiratory syndrome (SARS) outbreak in 2003 (28). The generally high perceived confidence in self-protection against COVID-19 in our study may be related to participants' high confidence in government control over the epidemic and high satisfaction with the dissemination of government information (29). More than 70% of the residents said that the people around them exhibited good protection for COVID-19. This indicated that most of the people still pay more attention to the protection during the post-lockdown period. Overall, the participants' attitudes toward COVID-19 were positive. When analyzing relevant factors about attitudes, some interesting features emerged. Gender, age, profession, marital status, and even education did not significantly influence attitude, which was not the same as the study conducted by Guy et al. (30). In an early survey of the KAP about COVID-19 in China, the attitude toward COVID-19 was significantly associated with gender, education level and occupation. This may be attributed to the different time periods of the three studies and the different tools used to assess the COVID-19 attitude. In previous studies, few researchers had used “whether living with susceptible people” as a variable. In view of the traditional concept of the Chinese family and the traditional virtues of the Chinese nation (“to respect the old and love the young”), we want to explore whether living with susceptible people will affect people's attitudes toward COVID-19. In fact, our study showed that the attitudes of people who live with elderly people, women, and children were much more positive. This may be because these people have the responsibility of caring for their families. This indicates that the government should pay more attention to the residents who do not live with elderly people, women, and children.

Most participants had good behaviors for managing COVID-19. Only 54.0% of the residents chose to take appropriate physical exercises at home to prevent the disease. Exercise could pay a protective role on people's mental health (31). Thus, people should be encouraged to exercise regularly. During the post-lockdown period, people began to eat out, and the Chinese government is currently encouraging the public to use serving spoons and chopsticks (20). However, almost half of the subjects did not follow these new behaviors. This is similar to a previous study (31). The eating habits of Chinese people are different from those of Westerners. They prefer to eat from the same plate, compared to Westerners using their own dishes to have food. Advocating the use of serving spoons and chopsticks is contrary to Chinese traditional customs (31). Thus, the government and the media need to further publicize the use of serving chopsticks and spoons. Binary logistic regression analysis about the practices showed that age, high level of attitude about COVID-19, and higher education were associated with better practices toward COVID-19. This study is consistent with the study by Alnasser et al., showing that the highest level of good practices regarding COVID-19 pandemic was of those aged 35–50, and of those with a bachelor's degree (32). The correlations between attitudes and practices had been well-established in various studies (16, 33). During the post-lockdown period of the epidemic, people's attitudes toward COVID-19 should be enhanced, the development of health education programs for young people be focused on, and the prevention and control of the epidemic for people with low education levels be paid attention to. Targeted health education according to different demographic characteristics should be taken to improve the prevention and control efforts.

## Limitations of the Study

This study had several limitations. First, convenience sampling was used in this study. Randomized sampling should be adopted in future studies to guarantee a better representation. Second, the online cross-sectional nature of study design limits the power of this research. Longitudinal research methods should be used in the future to obtain more information about the situation of the KAP for residents and the factors affecting the KAP toward COVID-19. The method of collecting data through the internet ignores those who cannot access the internet, such as older people, rural adults, and residents in remote areas with no network. Third, the participants in this study all came from urban communities in Shaanxi Province, while residents from rural areas were not included in the study. The KAP toward COVID-19 in rural residents may be different. Due to the fact that the participants in this study came from Shaanxi Province, which was not the area affected severely by COVID-19, the findings in this study were not generalizable for the residents who lived in other areas in China. Future studies should recruit a more representative and larger participant pool. We propose a comparative study involving rural areas and other districts. Despite the limitations described above, to our knowledge, there are limited studies about the KAP toward COVID-19 in China during the post-lockdown period.

## Conclusions

In conclusion, this study revealed that residents in Shaanxi Province have a good level of KAP toward COVID-19. Education was able to predict residents' knowledge toward COVID-19, while living with elderly people, women, and children predicted their attitude toward COVID-19. The better the level of attitude and the higher the educational background, the more positive the residents' practices. Thus, the health education programs be not only focused toward people with lower education, but also promoting the attitudes of residents, even during the post-lockdown period.

## Data Availability Statement

The original contributions presented in the study are included in the article/supplementary material, further inquiries can be directed to the corresponding author/s.

## Ethics Statement

The studies involving human participants were reviewed and approved by the Ethics Committee of Xi'an Medical University. The patients/participants provided their written informed consent to participate in this study.

## Author Contributions

NX and YZ: conceptualization. NX, NZ, GZ, and YZ: methodology. NX and XZ: software and formal analysis. XZ: validation. NX, XZ, FL, and YZ: investigation. YZ: resources and project administration. XZ and FL: data curation. NX: writing and original draft preparation. NX, ZG, and YZ: writing, review, and editing. YZ and GZ: supervision. All authors contributed to the article and approved the submitted version.

## Conflict of Interest

The authors declare that the research was conducted in the absence of any commercial or financial relationships that could be construed as a potential conflict of interest.

## Footnotes

^1^Tawalbeh LI, Al-Smadi AM, Ashour A, Alshajrawi A, Gammoh O, Abu-AL-Rous N. Public knowledge, attitudes and practice about COVID-19 pandemic. *J Public Health Afr*. (accepted). doi: 10.4081/jphia.2021.1519

## Abbreviations

COVID-19: officially declared as the coronavirus disease 2019
KAP: knowledge, attitude, and practices
OR: odds ratio
SD: standard deviation.
